# Hypermethylation of RASAL1: A Key for Renal Fibrosis

**DOI:** 10.1016/j.ebiom.2014.10.016

**Published:** 2014-10-28

**Authors:** Yuanjie Mao

**Affiliations:** Department of Biochemistry, National Cerebral and Cardiovascular Center Research Institute, Suita, Osaka 565-8565, Japan

Chronic kidney disease is a global epidemic that affects estimated 10% of the world population. As the final common pathway of chronic kidney diseases, renal fibrosis can result in massive destruction of normal kidney structure and subsequent impairment of the function. Activated fibroblasts are recognized to be principal mediators of renal fibrosis. Unlike in physiological wound repair, where fibroblast activation is spontaneously reversible, the fibroblast activation associated with renal fibrosis is perpetuated, and fibroblasts maintain their activated phenotype even when cultured in vitro (Müller and Rodemann, 1991, Rodemann and Müller, 1991).

Epigenetic modifications can cause the perpetuation of renal fibrosis. Previous studies have showed a prominent role of Ras signaling pathway in renal fibrosis (Hendry and Sharpe, 2003). Hypermethylation of RASAL1, encoding an inhibitor of the Ras protein, is associated with the perpetuation of fibroblast activation and experimental renal fibrosis (Bechtel et al., 2010). Transcriptional RASAL1 repression is associated with fibroblast activation in both physiological kidney repair and pathological kidney fibrosis. In physiological kidney repair, reversible fibroblast activation is associated with reversible RASAL1 suppression without RASAL1 hypermethylation; whereas in pathological kidney fibrosis, perpetual fibroblast activation is associated with irreversible RASAL1 expression due to RASAL1 promoter hypermethylation (Bechtel et al., 2010). Furthermore, this hypermethylation can be induced by long-term exposure to proinflammatory cytokines such as TGF-β1 (Bechtel et al., 2010). Bone morphogenic protein 7, an inhibitor of TGF-β1 signaling, normalizes the RASAL promoter hypermethylation and successfully inhibits experimental kidney fibrosis (Tampe et al., 2014).

In their article published in this issue of EBioMedicine, Tampe et al. demonstrated that the de-methylation of RASAL1 promoter induced by hydralazine is associated with ameliorating effects of experimental renal fibrosis. At the mechanistic level they demonstrated that hydralazine erases the aberrant RASAL1 promoter methylation mark and ameliorates experimental fibrogenesis by inducing a physiological mechanism of Tet3-mediated hydroxymethylation and subsequent replacement with unmethylated CpGs.

The results add new information to our current knowledge. First, by using transgenic mice harboring transgenes for doxycycline-inducible RASAL1 overexpression, RASAL1 over-expression was demonstrated to normalize the increased intrinsic proliferative activity of fibrotic fibroblasts in these mice with unilateral ureteral obstruction. Second, normalization of aberrant promoter methylation through administration of 5′-azacytidine or hydralazine is associated with attenuated fibroblast activation and fibrogenesis in experimental renal fibrosis. The key role of RASAL1 hypermethylation in renal fibrosis was further confirmed. Third, low dosage of hydralazine can attenuate renal fibrosis in rodent model whereas antihypertensive dosage of hydralazine cannot do so, possibly resulting from extra activation of Hif1α. And fourth, the levels of circulating methylated RASAL1 CpG promoter fragments reflect the degree of intrarenal RASAL1 promoter methylation and the extent of kidney fibrosis in animal models and in patients, similar to increased levels of methylated CpG fragments which can be detected in cancer patients.

Studying epigenetic changes in disease is important because these patterns of methylation are potentially reversible. This article implicated several messages which may add to clinical practice in the future. First, hydralazine at a low dosage could be a potential treatment for renal fibrosis. And second, circulating methylated RASAL1 promoter fragments is a possible biomarker for the severity of renal fibrosis.

Several questions remain to be addressed. First, the antifibrosis effect of RASAL1 overexpression is impressive. Epigenetic drugs available now such as azacitidine and decitabine or those currently being researched in vitro have major limitations, including lack of specificity and efficacy (Ptak and Petronis, 2008, Cantley and Haynes, 2013). The demethylation of hydralazine might modulate many downstream genes such as Hif1α and possibly cause additional adverse effects. In this regard, RASAL1 may be an optimal therapy target. Second, the effect of hydralazine in RASAL1 knocked-out mice of similar conditions remains unclear. Third, circulating methylated RASAL1 promoter fragments in peripheral blood corresponds with levels of intrarenal levels of RASAL1 promoter methylation and degree of fibrosis in kidney biopsies. However, the type of sample used for methylation association studies for disease has been a source of controversy. It has been suggested that epigenetic changes that cause disease are tissue specific. Differences in methylation patterns within tissues have been identified, and are larger between tissues in the same individual than within the same tissue between different individuals (De Bustos et al., 2009). Therefore, whether hydralazine de-methylation occurs in all types of cells is still unknown. And fourth, these results illustrate the importance in epigenetic control over immunity and the inflammatory response. The interaction of cytokines and hypermethylation of RASAL1 needs further investigation.

## Conflict of Interest

The author declares no conflicts of interest.
